# Leveraging a Simulated Patient Approach to Measure Pharyngitis Diagnostics and Prescribing in a Large Integrated Primary Care Network

**DOI:** 10.1017/ash.2025.243

**Published:** 2025-09-24

**Authors:** Anita Shallal, Michael Veve, Steven Fried, Christen Arena, Bruce Muma, Courtland Keteyian, Jodie Elsberg, Sharon Thomas, David Paculdo, Isabella Cooney, Czarlota Valdenor, Trever Burgon

**Affiliations:** 1 Henry Ford Hospital; 2 Henry Ford Hospital; 3 Henry Ford Medical Group; 4 Henry Ford Hospital, Wayne State University; 5 Henry Ford Health; 6 Henry Ford Health; 7 QURE Healthcare; 8 QURE Healthcare; 9 QURE Healthcare

## Abstract

**Background:** Bacterial pharyngitis is a commonly over-diagnosed ambulatory condition that can contribute to antibiotic overuse. Rapid antigen detection tests (RADT) are valuable in determining whether pharyngitis is caused by Group A streptococcus (GAS) and requires antibiotic therapy, or is viral in etiology. In 2021, Henry Ford Health partnered with QURE Healthcare to implement incentivized, evidence-based patient simulation training platforms for ambulatory primary care providers (PCP). This study aimed to describe outcomes of a simulated educational approach for ambulatory PCPs related to optimal pharyngitis testing and management. **Methods:** This was an IRB-exempt cross-sectional study of PCPs at an urban health system in Michigan. In 2024, four online simulated pharyngitis patients (two with characteristic GAS symptoms, two with hallmark viral symptoms) were incorporated into the program to assess antimicrobial stewardship among PCPs. PCPs provided care for simulated patients in random order over two seasons (spring and fall 2024), including the accuracy of medical decision-making about diagnostic testing and antibiotic treatment. At each decision point, PCPs received direct feedback on how decisions aligned with internal evidence-based guidelines. The primary outcome was to measure ordering decisions for RADTs and antibiotics by PCPs over the two simulation seasons. **Results:** 368 PCPs performed all four pharyngitis simulations. In cases where symptoms were congruent with GAS etiology, PCPs ordered RADT in 84.0%. Of those who ordered RADT, 98.7% ordered any antibiotic and 85.6% ordered an evidence-based antibiotic (i.e., penicillin or amoxicillin). For those who did not order RADT but received feedback within the case, 95.8% ordered any antibiotic and 76.3% ordered an evidence-based antibiotic. In cases with viral symptoms, 57.7% ordered RADT unnecessarily despite the low likelihood of GAS etiology. Antibiotics were ordered in 6.4% of cases with a negative RADT and without ordering RADT altogether. There was little difference in correct/incorrect RADT ordering patterns for the spring and fall seasons (P>0.05); there was an increase in ordering the preferred penicillin from the start of the first season to the end of the second season (25.0% to 33.7%, P = 0.062) and a 45.7% relative reduction in ordering non-recommended antibiotics (23.0% to 12.5%, P = 0.066). **Conclusion:** This study shows two-fold RADT challenges: overutilization for viral symptoms and underutilization for bacterial symptoms. Significant opportunities remain to increase guideline-recommended penicillin and reduce antibiotic use in viral pharyngitis cases. These results suggest that simulation-based measurement offers valuable insights into group-wide practice patterns and case-based feedback can improve evidence-based decision-making.

